# Cerebrasthenia the Bane of the American Brain-Worker

**Published:** 1905-01

**Authors:** E. Van Goidtsnoven

**Affiliations:** Atlanta, Ga.


### CEREBRASTHENIA THE BANE OF THE AMERICAN BRAIN-WORKER.

By E. VAN GOIDTSNOVEN, A.M., M.D.,

ATLANTA, GA.

I think I voice the sentiments of the medical profession when I state that cerebrasthenia is the bane of the American brain-worker.

To the least attentive observer of vital statistics and mortality tables the appalling fact stands aghast and unchallenged, that in the early and subsequent constant struggle, whether it be for honors and riches, or simply for an existence, twenty years of brain work, although possibly eventuating in the successful achievement of the desired end, but too often result in cerebral bankruptcy, in presenile debility, and in physical as well as in mental prostration.

Under those circumstances, virility ill-supported by a previous vigorous youth, is narrowed down to a few years of constant exhaustive struggle and prematurely merges into a pitiable condition of early and unseasonable decline and decay.

Sociological researches elicit the painful fact that men and women become gray-haired at twenty-five and white-haired at fifty. There hardly passes a day but we hear of a national calamity, notably the death of a comparatively young statesman, public benefactor, wealthy capitalist, famous orator, renowned politician, brilliant diplomat, promising man or woman of letters, wealthy manufacturer, honored warrior, etc. It would seem as though the valley of death was constantly yawning in our land for early victims of mental overwork.

Every steamer that leaves our shores carries off some dilapidated worshiper of the "*auri sacra fames*" to some gay capital of Europe, to the Mediterranean coast, to the Holy Land, to the lands made famous by Mahomet, Buddha and Confucius; and, but too often, the poor traveler returns home in a casket.

Cerebrasthenia, the great American disorder, simply means an exhaustion of brain force, and is largely the result of reflex irritations that take place not only through the ordinary and sensory nerves, but through the sympathetic system and vasomotor nerves. The reflex irritations may arise from any part of the body, but the chief centers of such irritations are the brain, the digestive system and the reproductive system.

The great volume of the brain, its complicated and artistic structure show it to be contrived for important ends. It is in constant communication through the spine, the cerebrospinal, the sympathetic system, and individualized little brains called ganglia, with every organ of the body; and thus vital force, motion, sensation and nutrition are incessantly imparted to the whole economy with due and specified control. On the other hand, disordered conditions arising in any of the distal or proximal areas or organs are afferently conveyed to the brain through reflex agency.

Cerebrasthenia, although wide-spread in this country, is not a modern disease. It has afflicted the human race from the beginning of history, the typical neurasthenic figures from the time of Job to the present day with his malnutrition, malassimilation, autoinfection and cell-exhaustion from mental strain and visceral perturbation.

Cerebrasthenia is a depraved state of the brain caused by malnutrition of its cells. It means a waste and inadequate restitution of that most important of brain food—*phosphorus*. Without phosphorus there is no thought, says Moleschott; and, according to Churchill, every kind of activity whether intellectual, emotional, locomotory or sensual, is conditioned either directly or indirectly by a wear of the phosphates, and whenever this activity exceeds a certain limit the organism is no longer in a normal state.

From all this is evident the immense importance of phosphorated pabulum in the general nutrition.

Once we understand the important role which the nervous system exercises over the animal economy in a state of health, it is easy to account for the disorders which a disturbed condition of the same in disease will have upon the various organs called into functional activity. Lithemia invariably presents in every typical case of neurasthenia with a full catalogue of symptoms which have led so many physicians astray in making a proper diagnosis and determining the differentiation between causes and effects.

Coincidentally with cerebrasthenia there is invariably present a condition of uricacidemia, of uric acid waste and by-products, incessantly generating toxic phenomena, perversion of function and organic disorders throughout the economy, either as an effect or a cause; and this constitutes the greatest stumbling-block in determining a true diagnosis as to what antecedent disorder ultimately resulted in brain exhaustion. To those who recognize a clinical and pathological relation between uric acid diathesis and rheumatism, gout, eczema, asthma, dyspepsia, diabetes, Bright's disease, cancer, etc., and finally, *cerebrasthenia*, *it is somewhat amazing that the majority of the medical profession, and especially the insurance companies, should restrict the importance of urine analysis to diabetic and albuminous excreta only.*

By way of illustration I wish to call the special attention to a type of disorder very common, very insidious in its evil doing, very lethal in its effects, and yet very much ignored not only by the laity, but by the medical profession: it is prostatic inflammation.

Reference is often made in medical literature to the analogy that exists between the womb and the prostate gland. In a practice extending over twenty-two years it has fallen to my lot to treat many cases of prostatic disorders. I am yet to see the first one that did not present hysterical manifestations, nervous disturbances occurring in paroxysms or simulating other diseases, attacks accompanied by an occasional abundant secretion of urine of low specific gravity, spasmodic stricture, etc. The converse will also frequently happen. For in prostatic enlargements there will often be found, as in hysteria, a perversion of certain faculties and characteristics of the patient, a weakness of the moral sense and of the will, an exaggeration of the emotions and of the affective faculties, with irregular and perverted action of the cutaneous, visceral and special senses. I simply mention this illustration as *e pluribus unum* selected from a labyrinth of records that have fallen under my observation and experience for nearly a quarter of a century. And I have no hesitancy in proclaiming that man has, at a certain and definite period of his existence, to undergo, *pari passu*, a change of life not unlike that of woman—affecting his generative organs—especially his prostate gland. It has often occurred to me that it is rather strange that so much special work has hitherto been entered into with regard to organs of females, whilst those of man have been so long ignored when attempting to relieve psychical disturbances, and I can not too strenuously insist upon the necessity of attending to the following rules:

1. It is the duty of every man engaged in professional or confining work to insure his brain against the insidious ravages which uric acid and its toxic by-products, such as xanthine, hypoxantine, creatine, tyrosine, etc., will inevitably inflict upon it, and finally end in *cerebrasthenia.*

2. He should ascertain not only incidentally, but with the regularity that imperious conditions demand, the nature of his disorder. His urine should be examined chemically and microscopically, qualitatively and quantitatively every three days during his ailment, and at least every three months after the evidence of the subsidence of the acute paroxysms, so as to guard against or keep up with the further lethal process.

It would be as impossible to estimate the enormous value of urine analysis as an aid to diagnosis, as it is impossible to appreciate the amount of suffering and the number of lives that have been saved through its instrumentality.

3. He must cut down the amount of albuminoids either eaten or drank, in order to reduce the demand upon the liver. The diet should be restricted to warm fluids, such as milk alone or with arrowroot, gruel, beef tea, etc. Milk and Apollinaris water constitute an excellent diet. Meat should be stricken out entirely from the dietary until the patient has made considerable improvement. Outdoor exercise should be enforced.

4. He must sweep away the waste from the blood by a cathartic and alterative, preferably at bedtime.

5. He must reduce the uric acid and its by-products into urea, and so get rid of them by flushing the circulation.

6. As all the forms of nervous exhaustion prepare the way for inebriety, and dipsomania is but too frequently the result of nerve degeneration, he should abstain from the use of alcoholic stimulants or at least reduce their daily use to the minimum. "A hereditary instability of nerve elements," says C. H. Hughes, "leads some organisms to irresistibly crave stimulants at certain times, generally after ordinary nervous and physical exhaustion, and these are satisfied with alcohol or opium. If they happen to find solace in opium readily, they become meconophagists, or if alcohol first falls in their way, and the insatiate longings of their unstable nervous organisms find in some beverage containing it the agreeable and temporarily satisfying impression their neuropathic organisms crave, their will (mastered by the lower dominant feeling) becomes a slave to the tyranny of a bad organism, regardless of consequences, and they enter, like the luckless de Quincy, into an Iliad of woes." Bearing in mind that, in the treatment of such cases, we should leave the nervous system in a state of almost physiological recuperability, the abrupt weaning process should not be for a moment entertained; and I most unqualifiedly and emphatically condemn it as unscientific and cruel, in view of the persisting morbid sequelæ of alcohol and opium addition. It is an inhuman and barbarous practice. The causation of cerebrasthenia is complex and mainly due in this country to opportunities and necessities of a rising civilization in a new and immense continent.

The indications for treatment furnished by nerve exhaustion are also complex, varied, and important. It is obvious enough that the measures indicated are those which will tend to restore the conditions of the blood and the nerves to the normal. The blood must be built up by an increase in the formation of the blood-corpuscles. The nerves should be refed and restored by a due supply of phosphorus in the shape of lecithin, phosphagon, nuclein, proto-nuclein, glycero-phosphates, or other forms of nerve-tissue builders, to be prescribed by the physician in charge, with due regard to each and every intercurrent disease that has preceded the prostration and been scrupulously *diagnosticated through urine analyses* and other means. Bearing in mind the specific poison which acted as a concomitant of a contributing disorder and, through impaired excretion, resulted in retained excreta upon the blood formation, it stands to reason that the cerebral cells can never regain their pristine power until the particular toxin has been successfully combatted and eradicated; and in the administration of a tonic and reconstructive following an intercurrent disease, one great point is to remember its causation, its progress and its stages before it reached its final outcome, *cerebrasthenia.*

"This, of course," in the language of G. M. Beard, the famous nerve-specialist, "necessitates and embraces a union of local and general medication, of mental therapeutics and hygiene, but no specific drug or treatment. Constitutional is relatively far more important than merely local treatment, and medicine more than surgery; although in some cases medicine and surgery need to unite their forces and work side by side."

I have written the above lines feeling, knowing and realizing that their practical application would find, as it were, an audience already prepared, or nearly so, to comprehend and believe and criticize and act upon their suggestions.

Years ago Horace Greely wrote a book on agriculture and headed it "What I know about farming." Having been a neurasthenic for more than twenty years, I claim I know something about cerebrasthenia: "*Elevê dans le sérail, j'en connais les détours.*" I have no specific remedy to offer. I simply wish to point out, for the benefit of my fellow sufferers a way to get out of the labyrinth of their woes. Many are the sinuous, winding paths, alleys, streets, avenues and boulevards that ultimately lead to that abode of doom and despair cerebrasthenia! This pathological, evolutionary and ultimate course and name should be known, traced up and pointed out. "*Experto crede.*"

Psychic influence in the practice of medicine can not be too much extolled. A conscientious physician takes cognizance of all psychic agencies and turns them to account in the treatment of disease. In my humble opinion there are two accomplishments which a medical man should most strenuously endeavor to obtain: Ability to diagnose pathological conditions and a systematic command and use of personal influence in piloting the frail bark of the afflicted, thus imitating the Great Physician when he said to Job:

"Gird up thy loins like a man."

*104 1-2 Whitehall.*

